# Closing the Gap in Global Neurosurgical Education via Online Conference: A Pre-Covid Survey

**DOI:** 10.7759/cureus.8015

**Published:** 2020-05-07

**Authors:** Simon R Downes, Tatiana Lykina

**Affiliations:** 1 Medicine, Oceania University of Medicine, Chicago, USA; 2 Allergy and Immunology, Oceania University of Medicine, Saint Petersburg, RUS

**Keywords:** neurosurgery, e-learning, medical education, online education, cme, covid-19

## Abstract

Introduction

A reliable network for peer review and feedback can lead to an increase in knowledge and improving patient care. As opportunities to participate in online continuing medical education (CME) increase, there is a reduction in the worldwide knowledge gap often due to a lack of resources to attend conferences and advanced training in person.

Methods

A total of 64 participants completed a 10-item anonymous online questionnaire to assess how their knowledge and applied practical skills improved by participating in online conferences, and whether this education modality adequately addresses challenges for countries with limited access to conferences or training.

Results

While an overall positive response toward this mode of neurosurgical education was expected, interesting insights were gained from the short-answer section, demonstrating a direct influence on clinical practice through online conference participation.

Conclusion

While limited in size, the study results support the expectation of a positive attitude toward neurosurgical e-learning, which translates directly to improving patient care and lessening the worldwide gap in neurosurgical education.

## Introduction

Physicians have numerous opportunities to update their clinical and academic knowledge by reading journals and attending training and conferences. However, physicians’ busy schedules present practical challenges for continuing medical education (CME) to improve medical knowledge and clinical skills. Consider, for example, a community-based physician with limited resources for traveling to an out-of-state conference who must instead rely on traditional forms of learning. As a result, this individual may not be as connected and proficient as colleagues located in large cities [1].

Even in the face of a pandemic with social distancing, learning must continue. Online conferencing and training overcomes geographical distance, and following the lockdowns due to coronavirus disease 2019 (COVID-19), in-person conferencing will never be the same.

Although online CME is relatively new, numerous studies have pointed to its efficacy by evaluating measurements of its effectiveness over time [2-8]. Moreover, there are expanding opportunities to connect physicians around the world utilizing various online platforms for neurosurgical education, such as live courses and webinars offered by the American Association of Neurological Surgeons.

While the advent of online neurosurgical education is difficult to ascertain, various web conferencing platforms have hosted education and communication between medical practitioners [9-12]. Therefore, it is important to point out that while participants of this study utilized one online platform as a source of online neurosurgical education, this platform may not be representative of all online neurosurgical education.

Although anecdotal evidence from neurosurgeons who have attended online conferences or presentations points to promising results, questions remain regarding the extent to which neurosurgeons benefit from online CME and whether it can supplant traveling to live conferences, particularly for those living in countries with limited access to advanced neurosurgical education.

Thus, this study addresses the following research questions:

1. Do neurosurgeons think that their knowledge and practical skills improve as a result of participation in online conferences?

2. Do neurosurgeons think that virtual neurosurgical education can provide opportunities when there is limited access to conferences or advanced training?

This study aims to understand the impact online neurosurgical education has on individual neurosurgeons, and also whether having access to online training and conferences will lessen the gap in global neurosurgery education. To explore the research questions, a multi-level questionnaire was created to measure attitudes toward the effectiveness of online learning through a combination of multiple-choice questions, Likert-scale questions, and a short answer fill-in section. Furthermore, demographic questions were added to better understand the population that completed the questionnaire.

Accordingly, to design questionnaire items that had a reasonable level of reliability or validity, we based our items on related scales which have been published in the literature [13-16].

## Materials and methods

Participants

In total, 64 individuals representing 23 countries participated in the questionnaire. Among the top countries, 14 (21.8%) participants were from Cameroon, 8 (12.5%) were from the U.S., and 5 (7.8%) each were from Pakistan and the Republic of the Congo; see Appendix 1 for a complete list of countries. Furthermore, participants ranged from 18 to 74 years of age, with the majority of participants between 25 and 34 (42.8%). The next largest age group was 35 to 44 (19.0%).

Procedure

We created an online invitation to complete a 10-item questionnaire. The questionnaire was anonymous, and no specific questions were related to stage of neurosurgery education or qualifications as a neurosurgeon. Respondents filled out the questionnaire between January 5, 2020 and February 3, 2020. To avoid selection bias, no incentive was offered to answer the questionnaire. Descriptive statistics were used to highlight the differences in respondents' opinions.

## Results

**Figure 1:**
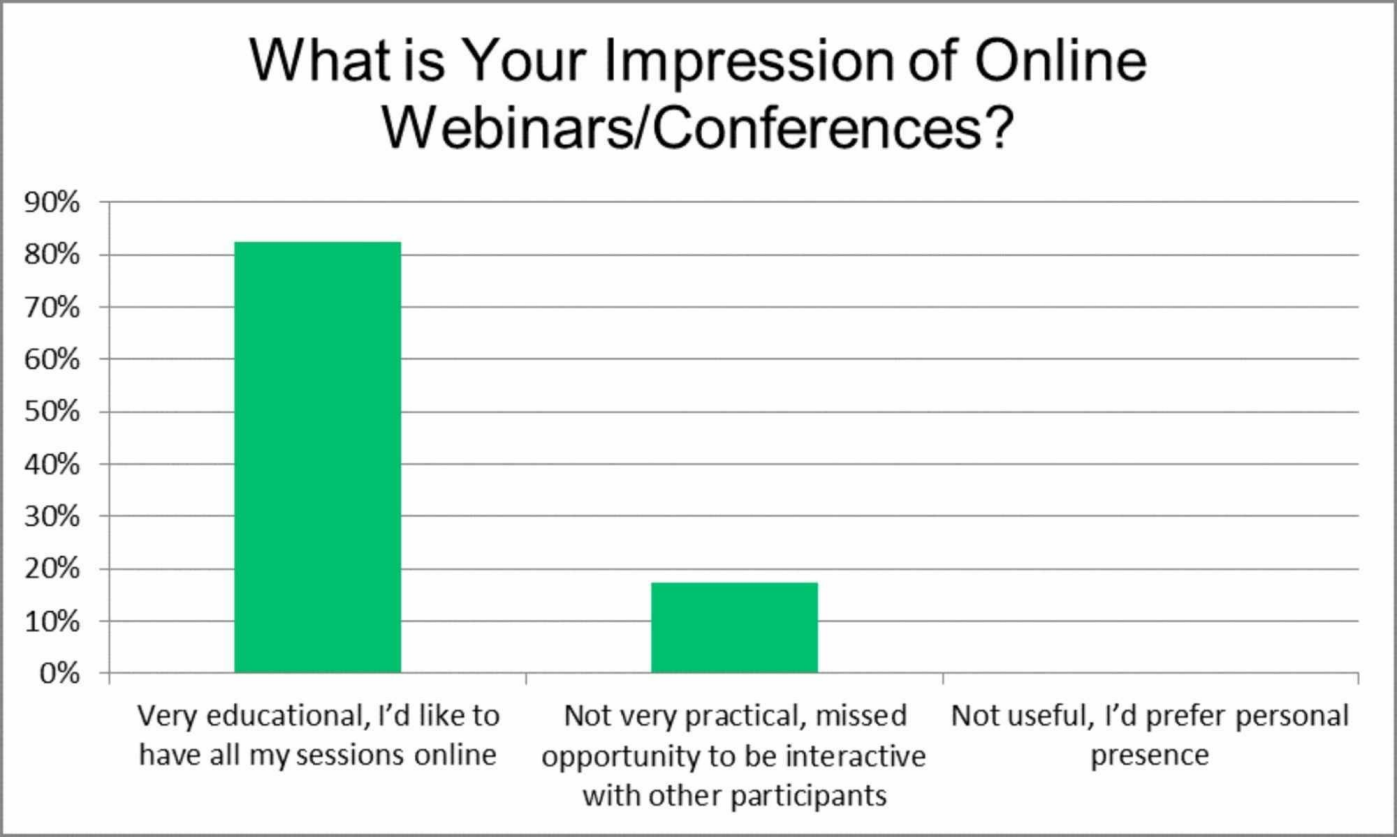
What is your impression of online webinars/conferences?

To gain an initial picture of participants’ attitudes toward online conferences, the first item asked, 'What is your impression of online webinars/conferences?' Most respondents (82.5%) answered positively, indicating they consider online sessions to be highly educational and would like to have their training sessions online, while (17.4%) did not consider online sessions to be useful and preferred to attend in person (Figure 1). Careful consideration is necessary here as these results apply to attitudes toward online education in general and not necessarily neurosurgical education.

**Figure 2:**
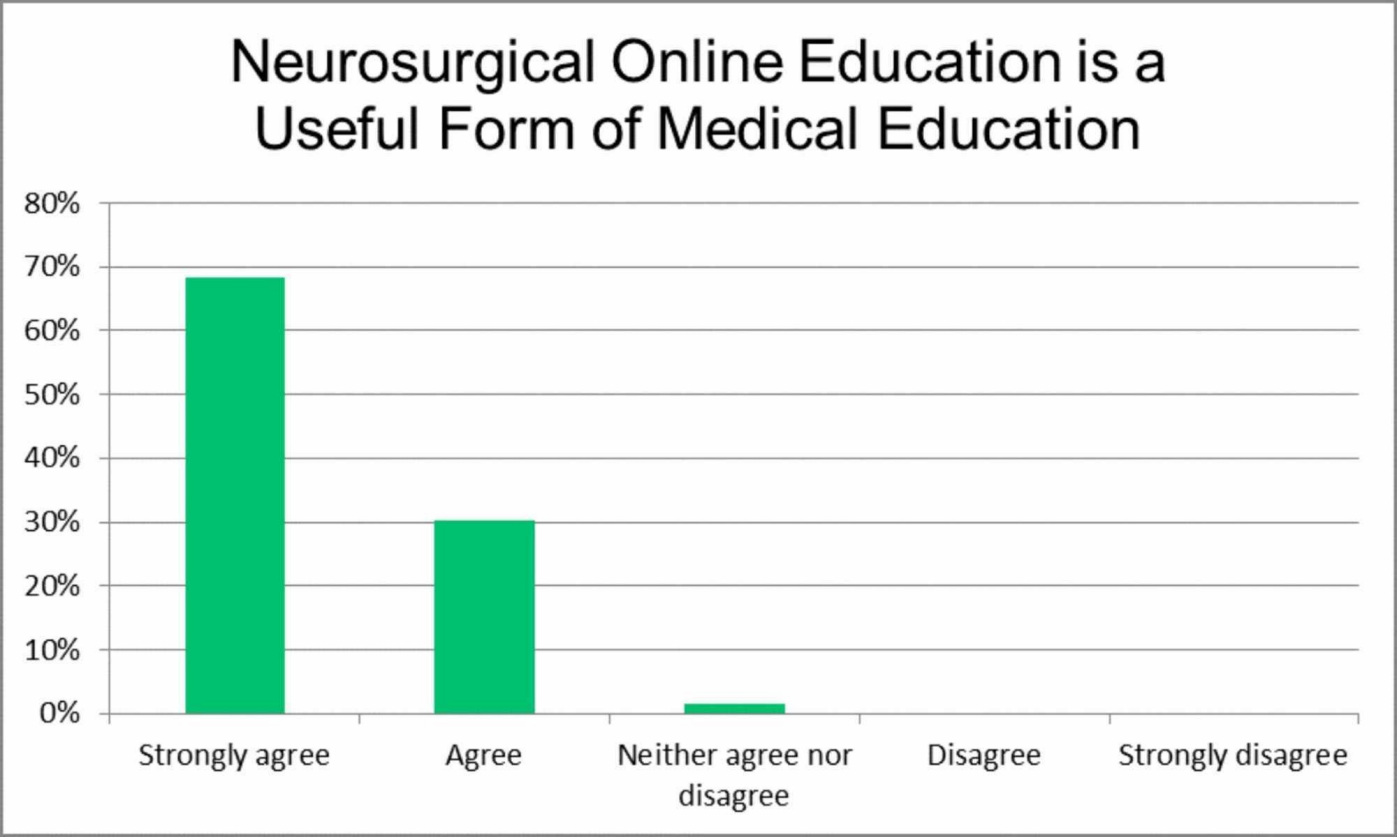
Neurosurgical online education is a useful form of medical education.

The second item, 'Neurosurgical online education is a useful form of education', aimed to determine how neurosurgeons value this novel form of education (Figure 2). Most (68.2%) strongly agreed or agreed (30.1%) that neurosurgical education in this format is useful.

**Figure 3:**
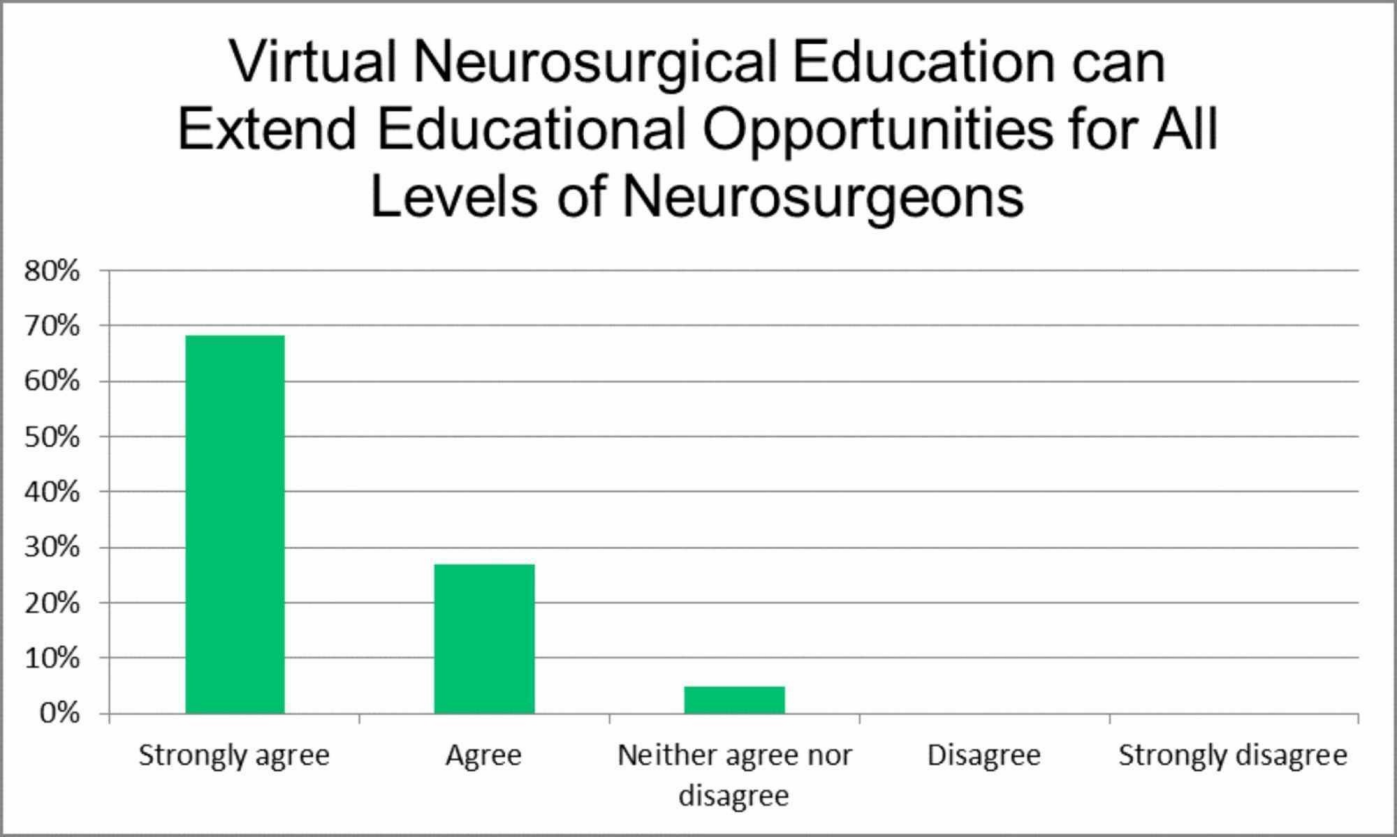
Virtual neurosurgical education can extend educational opportunities for all levels of neurosurgeons.

The third item, 'Virtual neurosurgical education can extend educational opportunities for all levels of neurosurgeons', considered whether a certain level of neurosurgical education is needed to derive a benefit from online sessions (Figure 3). Here, the majority of respondents strongly agreed (68.2%) or agreed (26.9%), signifying the opinion that online neurosurgical education is suitable at all levels of neurosurgery training.

**Figure 4:**
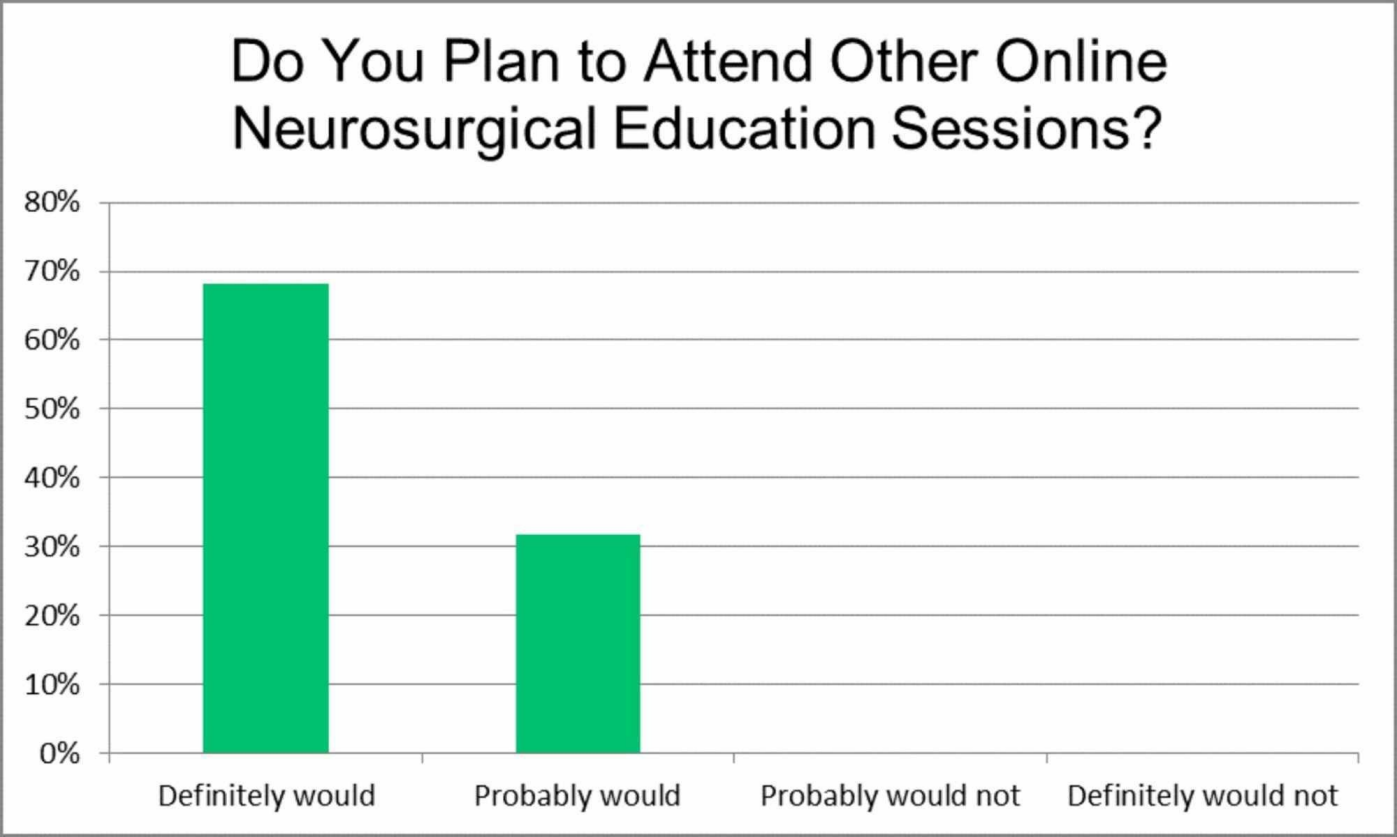
Do you plan to attend other online neurosurgical education sessions?

Next, item five, 'Do you plan to attend other online neurosurgical education sessions?', focused on individual intention for future participation in online neurosurgical education (Figure 4). Overall, 68.2% answered that they definitely would, while 31.7% answered they probably would attend other sessions. Given there were no negative responses, this could indicate a general acceptance of this form of online medical education.

**Figure 5:**
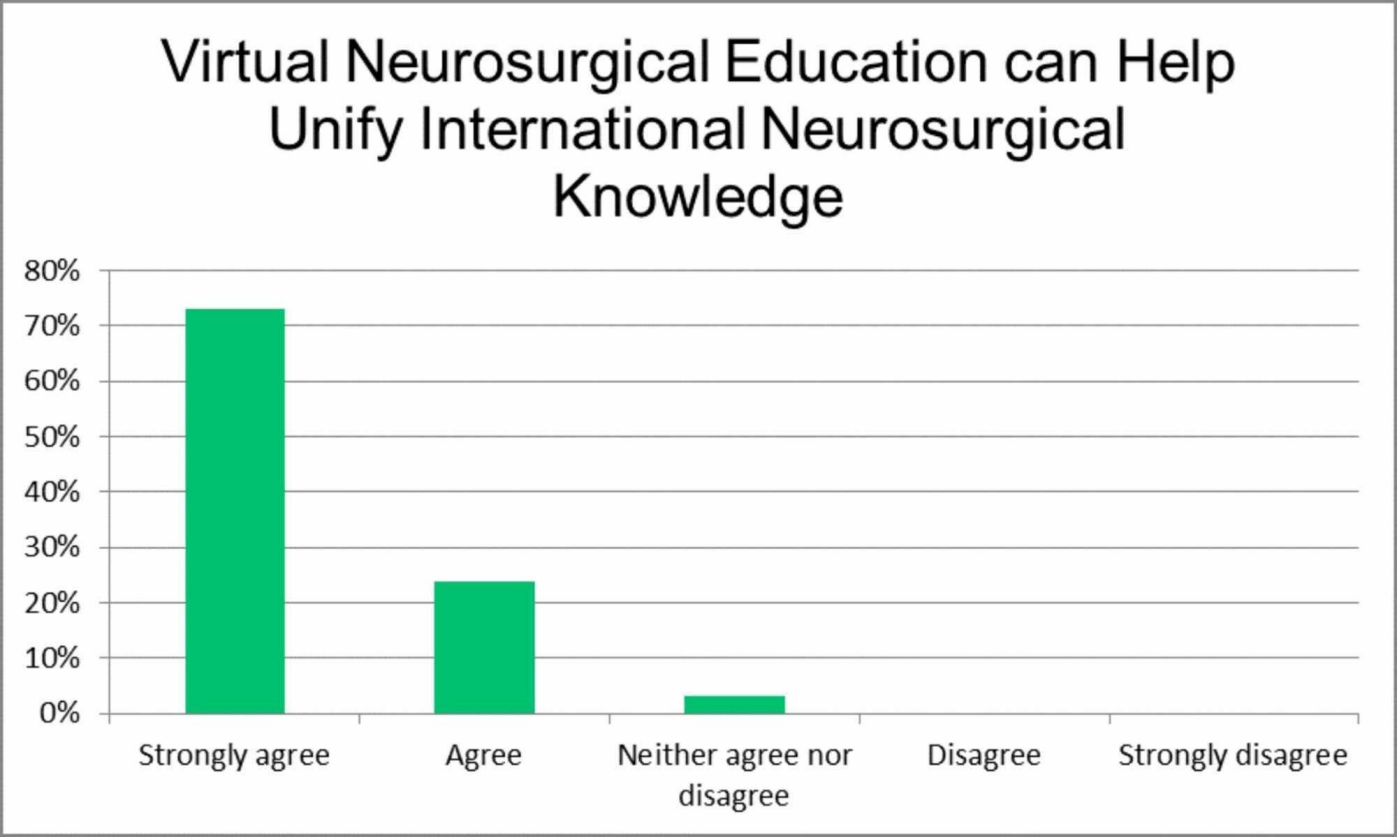
Virtual neurosurgical education can help unify international neurosurgical knowledge.

Item six, 'Virtual neurosurgical education can help to unify international neurosurgical knowledge', had participants consider whether this form of education can fill the global gap in education, particularly for developing countries where there are limited resources and those who cannot attend conferences in person due to cost or logistics (Figure 5). Most respondents either strongly agreed (73.0%) or agreed (23.8%).

**Figure 6:**
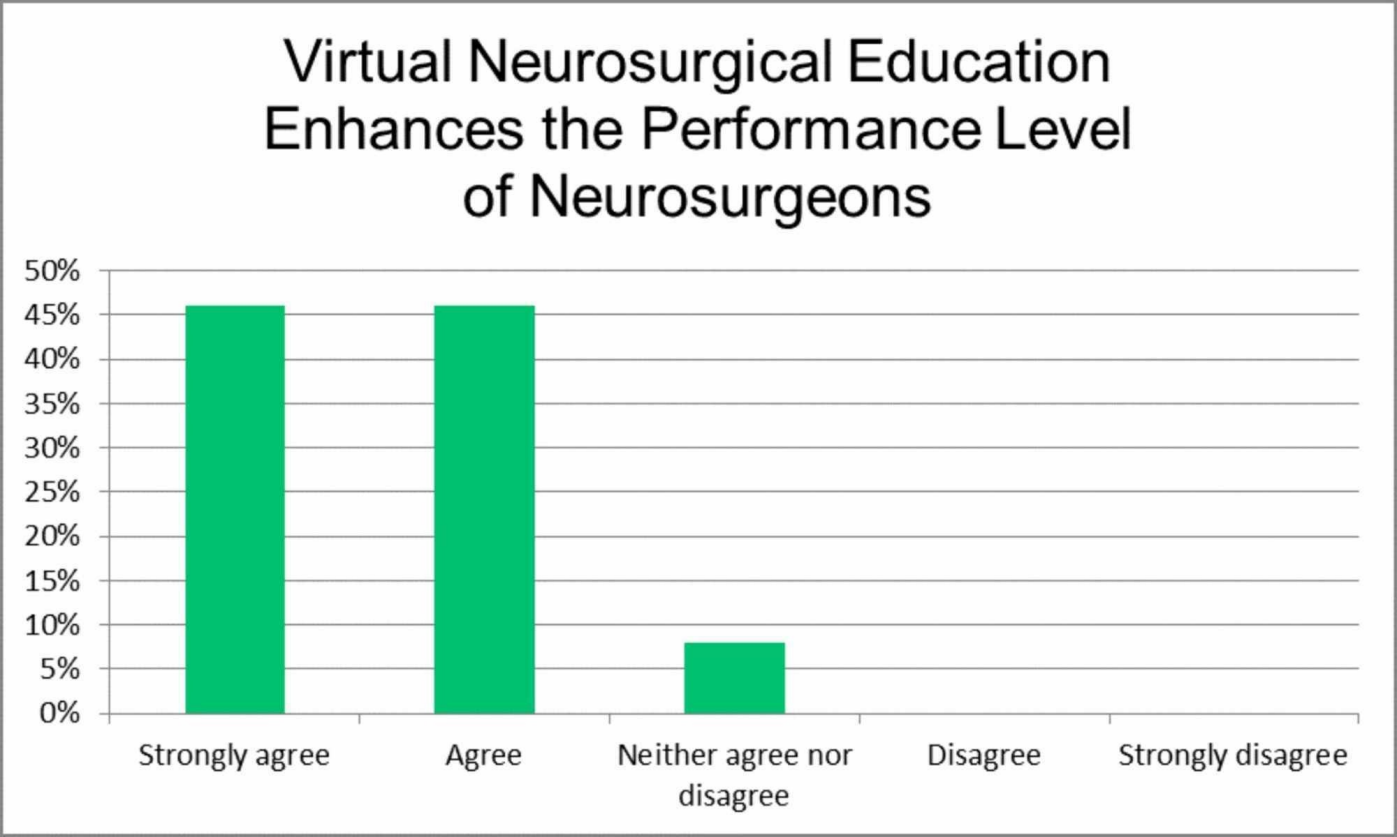
Virtual neurosurgical education enhances the performance level of neurosurgeons.

The seventh item, 'Virtual neurosurgical education enhances the performance level of neurosurgeons', shifts the focus to the practical implications of online neurosurgical education (Figure 6). While the majority strongly agreed (46.0%) or agreed (46.0%), some were undecided (7.9%). This presents an interesting question as to exactly what components of this form of education lead to an improvement of neurosurgical skills.

**Figure 7:**
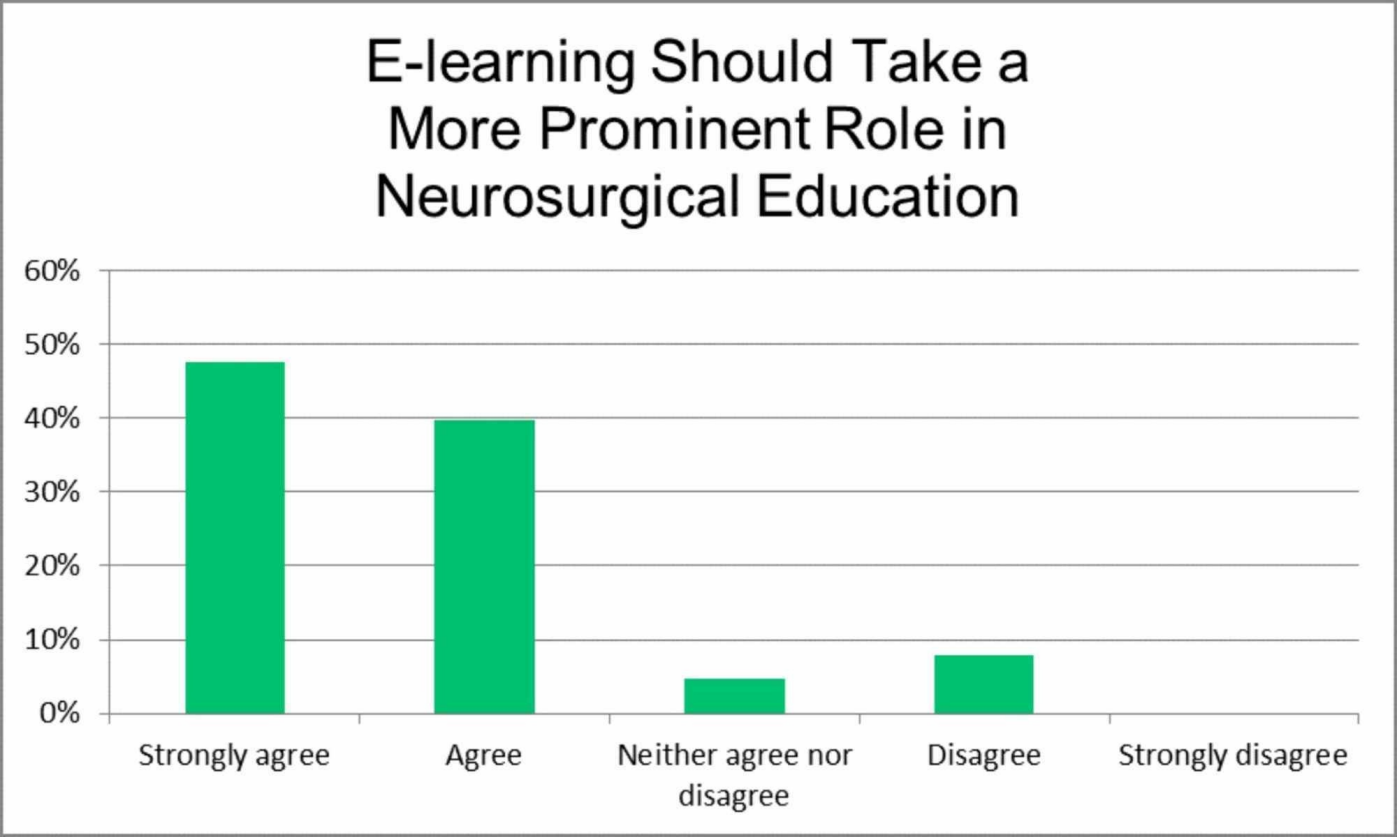
E-Learning should take a more prominent role in neurosurgical education.

Item eight, 'E-learning should take a more prominent role in neurosurgical education', inquired as to whether present neurosurgical education should place a greater emphasis on online education (Figure 7). Although most participants answered with strongly agree (47.6%) and agree (39.6%), this is the first instance where there was some disagreement (7.9%).

**Figure 8:**
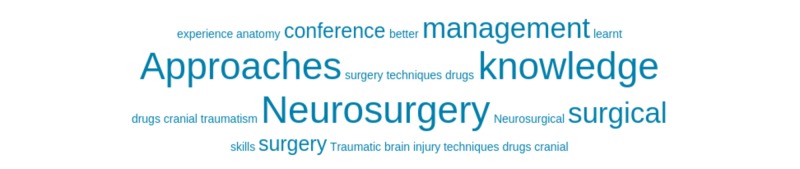
Word cloud for the question ‘Can you give specific examples of what you learned that you are going to take back to your practice to benefit patients?’

The final item, 'Can you give specific examples of what you learned that you are going to take back to your practice to benefit patients?’, asked respondents to write a short answer to demonstrate how their learning translated directly to their clinical practice. Figure 8 is a word cloud depicting the most commonly occurring words by respondents, while Appendix 2 shows the variety of responses by participants. It is important to note again that the questionnaire did not ask respondents about their level of neurosurgical training.

## Discussion

In this study, we investigated how the experience of attending online neurosurgical conferences and training affects knowledge and skills acquisition. The questionnaire results indicate that respondents generally had a positive attitude toward online conferences and training and found neurosurgical online education to be useful. Also, respondents noted they would attend other online neurosurgical education sessions in the future.

Most respondents agreed that online neurosurgical education can be beneficial for all levels of neurosurgeons and can help unify international neurosurgical knowledge. However, although most indicated that neurosurgeons’ performance level is enhanced by online neurosurgical education, not all agreed that e-learning should take a more prominent role in neurosurgical education.

Regarding the extent to which neurosurgeons believe online training will directly benefit patients, short-answer responses included improvement of surgical skills and approaches, better case management and decision-making for their clinical practice, and a clearer understanding of relevant anatomy and pathology.

The results indicate that there is a not only a positive attitude toward online neurosurgical education and direct benefits for neurosurgeons' clinical practice, but also that neurosurgeons agree this form of education may have wide-reaching possibilities for sharing knowledge.

Despite the small sample size, participants spanned 23 countries, suggesting an interest in online neurosurgical education on a global scale. However, a question arises as to whether practitioners-especially those in developing countries-who cannot afford to attend physical conferences may also face challenges in accessing online content such as live feeds, streaming, or group conferences.

The first limitation of this study is that given the length and scope of the questionnaire, additional factors could be investigated before drawing further conclusions. A further limitation is the anonymous nature of the questionnaire. While anonymity can increase the response rate, respondents cannot be contacted for follow-up regarding their responses.

Moreover, this study could not determine the respondents’ level of neurosurgical training. While one neurosurgical online education platform was targeted in this study, this may not be representative of the entire population of neurosurgeons.

Future studies should consider similar research questions but explore different methods, such as standardized scales to gauge the attitude of neurosurgeons toward online education. Additionally, a broader measurement may be needed to better understand the impact online neurosurgical education has on reducing the global education gap, particularly in developing countries.

## Conclusions

People around the world are increasingly sharing medical knowledge via the Internet in the wake of COVID-19. To stay connected and to continue learning, online conferences reduce geographic barriers and minimize the effects of social isolation. To expand medical knowledge and advance neurosurgical education, we must better understand the effectiveness and forms of online content delivery. This study points to a consensus that online neurosurgical conferences and training can contribute to closing the gap in global neurosurgical education. Further studies should look deeper into the impact of online neurosurgical education to suggest improvements for neurosurgical educators and all online medical education. In particular, it would be valuable to explore a variety of modalities with which online education reaches neurosurgeons and also how neurosurgeons access that information.

## Appendices

**Table 1:** Appendix 1

Country	Number of Respondents
Cameroon	14
United States	8
Pakistan	5
Republic of the Congo	5
India	3
Brazil	3
Venezuela	3
Indonesia	2
Lithuania	2
Panama	2
Mozambique	2
Albania	1
Angola	1
Ghana	1
Iraq	1
Ivory Coast	1
Morocco	1
Nigeria	1
Peru	1
Russia	1
UAE	1
Uruguay	1
Zimbabwe	1
No answer	1

**Table 2:** Appendix 2

ID	Date	Response
1	2/9/20	More knowledge about neurosurgery approaches
2	2/1/20	Microsurgical skills
3	1/31/20	Cisternostomy
4	1/31/20	Management of traumatic brain injury
5	1/31/20	Anatomy and approach
6	1/31/20	I must say they've been very educative and are having a positive impact on my study
7	1/31/20	Neuroanatomy, skills about diagnosis to management in neurosurgery
8	1/31/20	Cisternostomy
9	1/31/20	1-Management of traumatic brain injury, 2-Management of intracranial abscesses
10	1/31/20	Cisternostomy journal club
11	1/31/20	The surgery techniques and drugs about cranial traumatism
12	1/31/20	Neurological examination for a comatose patient, TBI management
13	1/31/20	Anatomy; pathology
14	1/31/20	Neurosurgical emergencies, neuroanatomy, health care economics
15	1/31/20	I learned how to properly assess the Glasgow Coma Score
16	1/31/20	New ways of approaching pathologies
17	1/27/20	Continuous storming of Basic knowledge that sometimes fade away
19	1/16/20	Endovascular neurosurgery better decision
20	1/11/20	Vascular neurosurgery
21	1/10/20	Video endoscopic spine surgery technics for foraminoplasty for example
22	1/10/20	Surgical techniques
23	1/9/20	Finding solution from case discussion
24	1/9/20	New surgical approach technique to apply in my daily practices
25	1/9/20	Only as reference in my medical practice
26	1/9/20	Imaging diagnosis for emergency neurological cases
27	1/9/20	Updated knowledge
28	1/9/20	Complication management
29	1/9/20	Lesson learned about brain trauma management during some conference
30	1/9/20	Skull base
31	1/9/20	My concepts on various topics of neurosurgery were quite helpful in my practice of neurosurgery
33	1/9/20	Aspects of base of skull surgery
ID	Date	Response
34	1/9/20	Manage of surgical treatment of cervical injuries
35	1/9/20	International experience. Would like to get CME for attending conferences
36	1/9/20	1) Enhance my general knowledge about neurosurgical items that are absence in our service 2) A good and cheap way for interactions with other colleagues around the world 3) Often a lot of colleagues with incredible high level of knowledge and experience
37	1/9/20	Images of actual anatomy are useful
38	1/9/20	Surgical skills
39	1/9/20	Workshops, observerships, conference with interaction in an area neurosurgery focused
40	1/9/20	Having an idea of the procedures help me with psychoeducation of the family which in turn diminishes their stress and increase patient engagement with post-surgery care. I'm cog rehab
41	1/9/20	Neurosurgical TV
42	1/9/20	Others experience
43	1/9/20	I began to do more cisternostomies in trauma patients
44	1/9/20	Neurology
45	1/9/20	Learning the different individual approaches to similar conditions. Improved knowledge base
46	1/8/20	Ischemic stroke, craniotomy, acute bony decompression
47	1/8/20	Principles of skull base surgery
48	1/8/20	I learnt to better manage a patient with TBI
49	1/8/20	Cisternostomy in TBI
50	1/8/20	Management of strokes, aneurysm, traumatic brain injury in low resource settings
51	1/8/20	Latest guidelines, surgical techniques, the don'ts of Neurosurgery, basics of Neurosurgery, brain and skull anatomy. Research, international collaboration. A view of the advanced world as we are from 3rd world country
52	1/8/20	More knowledge about neurosurgery approaches
